# Intraocular Pressure Fluctuation Throughout the Day

**DOI:** 10.7759/cureus.48826

**Published:** 2023-11-15

**Authors:** Veronica Noya-Padin, Jacobo Garcia-Queiruga, Belen Sabucedo-Villamarin, Noelia Nores-Palmas, Ricardo Taboada-Mecias, Eva Yebra-Pimentel

**Affiliations:** 1 Department of Applied Physics (Optometry Area), Universidade de Santiago de Compostela, Santiago de Compostela, ESP; 2 Department of Optometry, Health Research Institute of Santiago de Compostela (IDIS), Santiago de Compostela, ESP

**Keywords:** ocular risk factors, intraocular pressure fluctuation, intraocular pressure, glaucoma risk factors, canon tx-10

## Abstract

Purpose

To compare intraocular pressure (IOP) values at different time points, both in the total sample and according to iridocorneal angle aperture, to assess whether IOP fluctuations were constant throughout the day, and to examine correlations with other factors.

Methods

Over a single day, the IOP of 34 volunteers was measured at three-hour intervals from 9:00 a.m. to 6:00 p.m. To avoid any IOP value being affected by other measurements, anamnesis, slit-lamp evaluation (with iridocorneal angle measurement), and refractive status were performed after the final measurement. The differences between IOP values at different time points and IOP fluctuation at three-hour intervals were compared by ANOVA and Friedman test, respectively, both for the total group and according to iridocorneal angle aperture. For relationships, Pearson’s correlation was performed for parametric variables and Spearman’s correlation for nonparametric variables.

Results

Significant differences were observed in IOP between time points for the total sample (p < 0.001), but not for a narrow-angle group (p = 0.058). No significant differences were found in IOP fluctuations at three-hour intervals either in the total sample or according to angle aperture (all p ≥ 0.332). There was a positive correlation of IOP at different time points (all r ≥ 0.646, all p < 0.001) but no relationship with spherical equivalent, age, or sleep duration (all p ≥ 0.057). IOP at 12:00 p.m. was correlated with a 12:00 p.m. to 3:00 p.m. fluctuation (r = 0.428, p = 0.012); and IOP fluctuation between 9:00 a.m. and 12:00 p.m. was correlated with age (r = 0.485, p = 0.004).

Conclusion

As IOP decreases from morning until at least 6:00 p.m., measuring these two values during clinical evaluation is essential for the effective monitoring and prevention of IOP-related diseases.

## Introduction

Intraocular pressure (IOP) is an important parameter for the early detection of certain ocular pathologies, such as choroidal detachment or glaucoma [1,2]. Its usual distribution in the population ranges from 10 to 21 mmHg, with a mean value of approximately 15 mmHg in healthy adults [3]. Besides the absolute value, having knowledge of the daily fluctuations in IOP could help in evaluating the potential risk of ocular pathology development and making necessary treatment adjustments for patients with preexisting conditions [4,5]. The IOP fluctuation during the day can be because of circadian rhythms of blood and venous pressure, changes in cortisol levels, and the aqueous humor production and may even be affected seasonally [6]. It has been reported that those factors generate a physiological oscillation of 3 to 5 mmHg per day, which may be higher in pathological populations, even though IOP values are within the normal limits established for ocular health [7]. Previous studies found that this fluctuation appears to be cyclical, with the maximum IOP value occurring toward the end of the night period and early morning and the minimum value toward the end of the afternoon [8]. In addition to the above, other external factors can cause instantaneous fluctuations in IOP, such as body position, with IOP increasing in the supine position compared to more upright positions [9]. This fact has led some studies to conjecture that the supine body position during sleep may be a possible contributor to the nocturnal IOP increase, although the findings remain inconclusive [10]. The aims of the present study were as follows: 1) to compare IOP values at different times in the total sample, 2) to compare IOP values at different times by classifying the participants according to the iridocorneal angle opening, 3) to assess if IOP fluctuations were constant throughout the day, and 4) to examine the correlations between IOP and its fluctuation with age, refractive error, and sleep time the previous night.

## Materials and methods

Participants

The PS Power and Sample Size Calculations Software Version 3.1.2 (Copyright © by William D. Dupont and Walton D. Plummer, Nashville, TN) was used to calculate the sample size. Based on previous research [11], it was determined that the mean standard deviation (SD) of repeated IOP measurements is normally distributed with a value of 5 mmHg. To have 90% power at a significance level of α = 0.05 (type I error associated) with a confidence level of 95% to detect a minimum difference of 2 mmHg, the minimum number of participants required was 21. To achieve a more reliable study, a larger population was recruited, including a sample of 35 participants (12 men and 23 women) with a mean age of 34.7 ± 16.74 years (range: 18-65 years), recruited from subjects attending the Optometry Clinic of the Universidade de Santiago de Compostela (Galicia, Spain). Inclusion criteria included a spherical equivalent error between -8.00 to +4.00 D, absence of previous diagnosis of glaucoma and not having undergone ocular surgery. All participants were informed of the procedures and provided their written informed consent to be included in the study. The study protocol followed the guidelines outlined in the Declaration of Helsinki and received approval from the Institution’s Ethics Committee (CEIG 2013/360).

Study design

Over the course of a single day, the IOP of both eyes was measured on four occasions (at 9:00 a.m., 12:00 a.m., 3:00 p.m., and 6:00 p.m.), always after resting for 15 minutes in a seated position [5,12,13]. The measurements were also performed in the sitting position by the noncontact tonometer Canon TX-10 (Canon Inc., Tokyo, Japan), which determines the mean IOP value automatically by taking three measures of the patient’s cornea deformation when blowing an air pulse [14]. The tests conducted to confirm compliance with the inclusion criteria were performed after the last IOP measurement at 6:00 p.m. to avoid the possibility that IOP values could be influenced by other measurements. These tests were anamnesis, slit-lamp examination using the Topcon SL-D4 device (Topcon Corporation, Tokyo, Japan), and refractive error measurement by subjective refraction with the fogging technique performed by an optometrist. Age, gender, ocular, and systemic diseases, previous ocular surgeries, and time slept the previous night were asked during the anamnesis. Additionally, an estimation of the iridocorneal angle value was calculated using the Van Herick slit-lamp technique, classified as grade four if the distance between the cornea and the iris was equal to or greater than the slit-lamp thickness of the corneal section, and grade less than four for smaller distances [15,16]. Finally, the subjective refractive condition was measured.

Statistical analysis

SPSS statistical software v. 25.0 for Windows (IBM SPSS Statistics for Windows, Armonk, NY) was used for data analyses. Significance was set at p ≤ 0.05 for all the tests. As IOP is a parametric variable [3], the analysis of variance (ANOVA) test for dependent samples was used to assess the differences between IOP values at different times. Sphericity was evaluated with Mauchly’s test and, if necessary, the appropriate corrections were applied in each case using the Greenhouse-Geisser or Huynh-Feldt correction [17]. Additionally, the Sidak test was used to detect significant pairwise differences. This analysis was performed twice, first to assess IOP differences in the total sample (Objective 1) and then to assess IOP differences when participants were grouped according to the classification obtained in Van Herick’s technique (group 1: Van Herick < 4; group 2: Van Herick = 4) (Objective 2).

The differences between IOP fluctuations in three-hour ranges were also evaluated (i.e., if fluctuation between 9:00 a.m. and 12:00 a.m. was different from that observed between 12:00 a.m. and 3:00 p.m. or between 3:00 p.m. and 6:00 p.m.), using the Friedman test, because, after assessing the normality of the distribution with the Shapiro-Wilk test, it was observed that the differences in the absolute value of differences are nonparametric variables (Objective 3).

Finally, the relationship between the variables asked during the anamnesis, refractive condition, and IOP or IOP differences were evaluated. Pearson’s correlation test was used to evaluate the relationship between IOP values and refractive condition (as spherical equivalent); other correlations (IOP fluctuation, age, or time asleep) were performed with Spearman’s correlation test (Objective 4). Correlation between variables was described as weak (0.20-0.40), moderate (0.41-0.60), good (0.61-0.80), or strong (0.81-1.00).

## Results

Based on the preestablished inclusion criteria, the final sample consisted of 34 participants (12 men and 22 women), with a mean age of 34.2 ± 16.74 years (range: 18 to 65 years). One participant was excluded from the analysis because of having a spherical equivalent error greater than +4.00 D. The mean spherical equivalent refraction was -1.00 ± 1.81 D (range: -7.00 to +2.38 D), with 24 participants having a Van Herick’s value of four (all cases in both eyes) and 10 participants having a Van Herick’s value of less than four.

**Figure 1:**
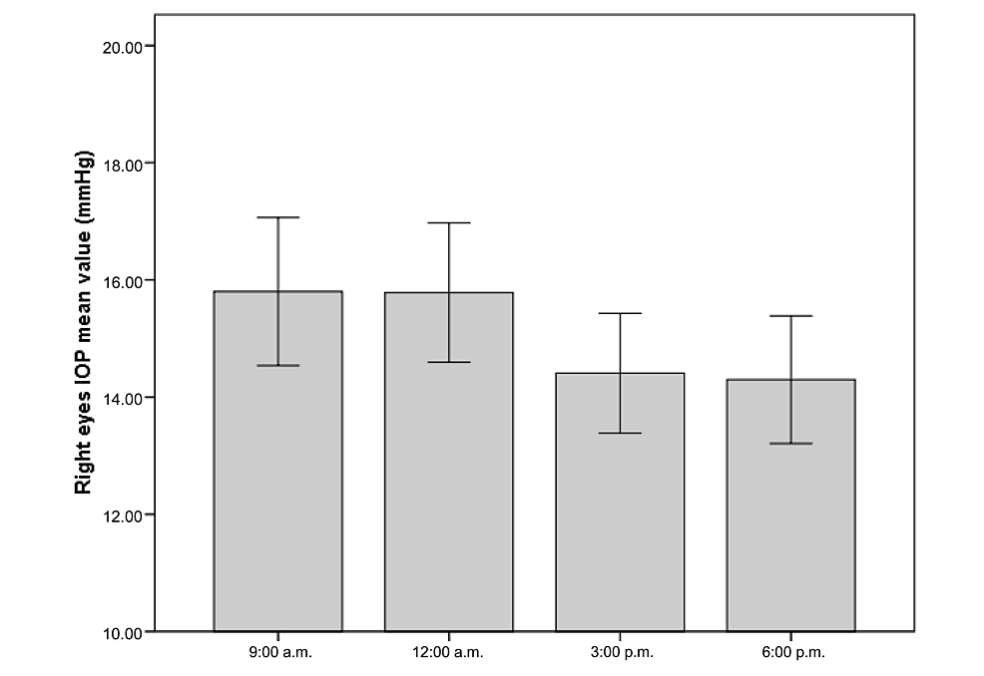
Mean IOP ± SD values in the right eyes of participants at the different time points. n = 34; IOP = intraocular pressure; mmHg = millimeters of mercury. Error bars represent the 95% confidence interval.

Regarding IOP, the mean IOP value at 9:00 a.m. for the right eyes was 15.8 ± 3.62 mmHg (range: 9.8 to 28.4), and, for the left eyes, it was 15.2 ± 3.73 mmHg (range: 8.7-25.7). No significant differences were found between both eyes (paired t-test, p = 0.053). Therefore, statistical analyses were performed only on the right eye of each participant to avoid statistical overestimation associated with the combined analysis of both eyes [18]. Thus, it was obtained that the mean IOP for the right eyes at 12:00 a.m. was 15.8 ± 3.41 mmHg (range: 9.1 to 26.6), at 3:00 p.m. it was 14.4 ± 2.93 mmHg (range: 9.7 to 20.8), and at 6:00 p.m. it was 14.3 ± 3.12 mmHg (range: 8.7 to 20.0) (Table 1) (Figure 1).

**Table 1:** IOP values of the right eyes and differences between measurement points for the total sample. All values in mmHg. AM = ante meridiem; IOP = intraocular pressure; mmHg = millimeters of mercury; PM = post meridiem; SD = standard deviation; 95% LoAs = 95% limits of agreement; 95% CI of LoAs = 95% confidence interval of limits of agreement. Significant differences (p ≤ 0.050) are indicated by (*).

Hour	Mean IOP ± SD	Mean IOP difference ± SD	p	95 % LoAs		95 % CI of LoAs	
				Lower	Upper	Lower	Upper
9:00 a.m.	15.8 ± 3.62	0.02 ± 0.368	1.000	-0.70	0.74	-0.56/-0.97	0.60/1.01
12:00 a.m.	15.8 ± 3.41						
9:00 a.m.	15.8 ± 3.62	1.39 ± 0.424*	0.014	0.56	2.22	0.72/0.25	2.06/2.53
3:00 p.m.	14.4 ± 2.93						
9:00 a.m.	15.8 ± 3.62	1.50 ± 0.451*	0.013	0.62	2.38	0.79/0.29	2.21/2.71
6:00 p.m.	14.3 ± 3.12						
12:00 a.m.	15.8 ± 3.41	1.38 ± 0.464*	0.033	0.47	2.29	0.65/0.13	2.11/2.63
3:00 p.m.	14.4 ± 2.93						
12:00 a.m.	15.8 ± 3.41	1.49 ± 0.427*	0.009	0.65	2.33	0.81/0.34	2.17/2.64
6:00 p.m.	14.3 ± 3.12						
3:00 p.m.	14.4 ± 2.93	0.11 ± 0.354	1.000	-0.58	0.80	-0.45/-0.84	0.67/1.06
6:00 p.m.	14.3 ± 3.12						

Differences between IOP values in the total sample

Statistically significant differences were observed in IOP values between the measurement points (Mauchly’s W indicates no violation of the sphericity assumption, p = 0.224; p < 0.001). Pairwise comparisons are reported in detail in Table 1. On the paired analysis, significant differences were found in IOP values at 9:00 a.m. compared to 3:00 p.m. and 6:00 p.m. and at 12:00 a.m. compared to 3:00 p.m. and 6:00 p.m.

Differences between IOP values based on the iridocorneal angle

When the sample was divided according to the iridocorneal angle (Van Herick less than or equal to 4), significant differences were found in the group with a Van Herick angle equal to four (Mauchly’s W indicates no violation of the sphericity assumption, p = 0.626; p < 0.001), while not in the group with a Van Herick angle less than four (Mauchly’s W: p = 0.049; ε = 0.679; Greenhouse-Geisser, p = 0.058). Pairwise comparisons are reported in detail in Table 2.

**Table 2:** IOP values of the right eyes and differences between measurement points as a function of the Van Herick angle value of the volunteers. All values in mmHg. n = 34; AM = ante meridiem; IOP = intraocular pressure; mmHg = millimeters of mercury; PM = post meridiem; SD = standard deviation; 95 % LoAs = 95 % limits of agreement; 95 % CI of LoAs = 95 % confidence interval of limits of agreement. Significant differences (p ≤ 0.050) are indicated by a (*).

	Hour	Mean IOP ± SD	Mean IOP difference ± SD	p	95% LoAs		95% CI of LoAs	
					Lower	Upper	Lower	Upper
Van Herick < 4 (n = 10)	9:00 a.m.	17.0 ± 4.86	-0.62 ± 0.885	0.985	-2.35	1.11	-2.02/-3.00	0.78/1.76
	12:00 a.m.	17.6 ± 3.79						
	9:00 a.m.	17.0 ± 4.86	1.33 ± 1.011	0.777	-0.65	3.31	-0.27/-1.38	2.93/4.04
	3:00 p.m.	15.7 ± 3.71						
	9:00 a.m.	17.0 ± 4.86	1.90 ± 0.978	0.409	-0.02	3.82	0.35/-0.73	345/4.53
	6:00 p.m.	15.1 ± 3.06						
	12:00 a.m.	17.6 ± 3.79	1.95 ± 1.107	0.510	-0.22	4.12	0.20/-1.02	3.70/4.92
	3:00 p.m.	15.7 ± 3.71						
	12:00 a.m.	17.6 ± 3.79	2.52 ± 0.808	0.072	-0.94	4.10	1.24/0.35	3.80/4.69
	6:00 p.m.	15.1 ± 3.06						
	3:00 p.m.	15.7 ± 3.71	0.57 ± 0.484	0.848	-0.38	1.52	-0.20/-0.73	1.34/1.87
	6:00 p.m.	15.1 ± 3.06						
Van Herick = 4 (n = 24)	9:00 a.m.	15.3 ± 2.95	0.28 ± 0.370	0.973	-0.45	1.01	-0.31/-0.71	0.87/1.27
	12:00 a.m.	15.0 ± 3.00						
	9:00 a.m.	15.3 ± 2.95	1.42 ± 0.446*	0.024	0.55	2.29	0.71/0.22	2.13/2.62
	3:00 p.m.	13.9 ± 2.45						
	9:00 a.m.	15.3 ± 2.95	1.34 ± 0.503	0.082	0.35	2.33	0.54/-0.01	2.14/2.69
	6:00 p.m.	14.0 ± 3.15						
	12:00 a.m.	15.0 ± 3.00	1.14 ± 0.478	0.146	0.20	2.08	0.38/-0.14	1.90/2.42
	3:00 p.m.	13.9 ± 2.45						
	12:00 a.m.	15.0 ± 3.00	1.05 ± 0.486	0.221	0.10	2.00	0.28/-0.26	1.82/2.36
	6:00 p.m.	14.0 ± 3.15						
	3:00 p.m.	13.9 ± 2.45	-0.08 ± 0.459	1.000	-0.98	0.82	-0.81/-1.31	0.65/1.15
	6:00 p.m.	14.0 ± 3.15						

Additionally, the differences between the IOP value of eyes with a Van Herick equal to four versus those with a grade lower than four were also assessed. No significant differences were found at any time of the day (paired t-test, all p ≥ 0.158).

Differences between IOP fluctuations in three-hour intervals

No significant differences were found in IOP fluctuations in three-hour intervals (Friedman test, all p ≥ 0.571). Similarly, no significant differences were observed when the sample was analyzed separately according to the Van Herick value (Friedman test, all p ≥ 0.332).

Correlation between IOP values and other factors

There was a good positive correlation between the IOP values at different times (Pearson’s correlation, all r ≥ 0.646, p < 0.001). No significant relationships were found between IOP at any measurement point and spherical equivalent (Pearson’s correlation, all p ≥ 0.064), age (Spearman’s correlation, all p ≥ 0.057), or time slept the night before (Spearman’s correlation, all p ≥ 0.103). Regarding correlations between IOP at the different measurement times and IOP fluctuation, there was only a statistically significant correlation between IOP at 12:00 a.m. and IOP fluctuation from 12:00 a.m. to 3:00 p.m. (Spearman’s correlation, r = 0.428, p = 0.012); the other values were not correlated (Spearman’s correlation, all p ≥ 0.144). A significant moderate positive correlation between age and IOP fluctuation during the period of 9:00 a.m. to 12:00 a.m. was also observed (Spearman’s correlation, r = 0.485, p = 0.004). No significant relationships were found between age and IOP fluctuation during other periods of the day (Spearman’s correlation, all p ≥ 0.751).

## Discussion

IOP is a parameter used for the detection, diagnosis, and follow-up of ocular pathologies such as glaucoma [1]. However, this parameter fluctuates throughout the day [4]. This means that even if a subject’s IOP is within the normal range (between 10 and 21 mmHg) at a certain time, it could exceed the umbral value of 21 mmHg when measured at another time of the day. As a result, a subject may appear normotensive when, in fact, they experience ocular hypertension at certain times of the day. In the present study, the fluctuation of IOP throughout the day was evaluated, and the possible relationships between these fluctuations and other factors such as spherical equivalent, age, or time slept the night before were assessed.

Similar to previous reports, it was found that there is no significant difference between the IOP of both eyes [19]. Furthermore, also in accordance with previous findings [20], the results of this study showed that IOP fluctuates throughout the day, being its highest value in the morning (15.8 ± 3.62 mmHg at 9:00 a.m.) and decreasing as the hours pass (14.3 ± 3.12 mmHg at 6:00 p.m.). Some research claims that the repeatability of the IOP curve over time is not preserved in either healthy or glaucomatous eyes [21,22]. However, many reports agree on the daily pattern of IOP [20,23], and there is also research that finds good repeatability in this pattern [24].

When evaluating the differences in IOP according to the Van Herick angle of the subjects, significant differences were found only in the group of Van Herick equal to four, specifically for the period from 9:00 a.m. to 3:00 p.m., but no differences were observed in the group with lower iridocorneal angle. These results are contradictory to previous literature [25,26], where fluctuations were found in both groups and especially in the subjects with a narrower angle (although these were pathological subjects diagnosed with glaucoma) [27]. It is possible that the present finding is because of the small sample size, as the Van Herick less than four group had only 10 participants, compared to 24 participants in the Van Herick grade four group.

As for the differences between fluctuations, no differences were found between the different periods either in the total sample or when separating according to the Van Herick angle, indicating that the fluctuation rate is similar throughout the day.

Finally, regarding relationships, in the present study, no significant correlation was found between IOP and age or spherical equivalent, which differs from previous research [28], nor with the time slept the night before the measurements or IOP fluctuation between different periods of the day. These differences may be because of the sample size. There was also no relationship between IOP fluctuation and age, as observed by Arora et al. [23], nor with the hours of sleep or refractive error. This aligns with prior research findings, which indicated a comparable level of diurnal variation regardless of refractive error in emmetropes and myopes [29].

It can be stated that it is difficult to compare IOP fluctuations across different studies because of the numerous factors that can influence its value both instantaneously and long-term factors and because of the different measurement techniques [23].

One of the strengths of the present study is that the measurements for each subject were taken on the same day. In terms of limitations, the study sample was small, specifically in relation to subgroups classified by iridocorneal angle, 24-hour follow-up of IOP was not performed, and overnight IOP measurements were not taken. These measurements would have been useful to detect the actual timing of the nocturnal IOP peak. Additionally, no dietary restrictions were imposed between IOP measurements, and as mentioned in the introduction section, activities such as water intake could affect the IOP value.

## Conclusions

Despite the challenges and costs of monitoring IOP throughout the day, the results of this study along with existing literature, emphasize the importance of measuring IOP at different times to grasp its dynamics in each subject fully. As IOP tends to peak in the morning and gradually decreases until at least 6:00 p.m., it is essential to include these two measurements in clinical assessment for effective monitoring and preventing IOP-related diseases.
